# The Structural Fingerprint of Therapeutic Monoclonal Antibodies Determined Using a Combination of Near-UV Circular Dichroism and Statistical Approach for Comparative Analysis

**DOI:** 10.1021/acs.analchem.5c00718

**Published:** 2025-06-10

**Authors:** Masato Kiyoshi, Taiji Oyama, Hiroko Shibata, Satoko Suzuki, Yuji Higuchi, Kouhei Tsumoto, Akiko Ishii-Watabe

**Affiliations:** † Division of Biological Chemistry and Biologicals, National Institute of Health Sciences, 3-25-26 Tonomachi, Kawasaki-ku, Kawasaki, Kanagawa 210-9501, Japan; ‡ 133823 JASCO Corporation, 2967-5 Ishikawamachi, Hachioji, Tokyo 192-8537, Japan; § Department of Bioengineering, School of Engineering, The University of Tokyo, 7-3-1 Hongo, Bunkyo-ku, Tokyo 113-8656, Japan; ∥ The Institute of Medical Science, The University of Tokyo, 4-6-1 Shirokanedai, Minato-ku, Tokyo 108-8639, Japan

## Abstract

The higher-order structure (HOS) of therapeutic monoclonal antibodies is one of the important quality characteristics in terms of efficacy and safety. However, the HOS of therapeutic monoclonal antibodies has been assessed using low- to medium-resolution spectroscopy. Furthermore, because the analysis of the obtained spectra has been performed by visual evaluation in many cases, their specificity, structural similarity, and statistical significance have not been thoroughly discussed. Therefore, there is an increasing need for advanced, statistically comparable, and highly specific analytical methods for the HOS of therapeutic monoclonal antibodies. Herein, we describe an analytical method for the HOS of therapeutic monoclonal antibodies using a powerful combination of near-UV circular dichroism (CD) and statistical analysis. The obtained near-UV CD spectra of 14 therapeutic monoclonal antibodies were employed for principal component analysis. Moreover, the spectral data were converted into Euclidean distances to perform equivalence tests and significance tests. The results clearly demonstrated that each antibody had a unique near-UV CD spectrum, like a structural fingerprint. All antibodies were judged to be not equivalent to other antibodies and were also judged to be significantly different from other antibodies. Moreover, the equivalence tests were performed on several lots of antibodies, and each lot of the antibodies were judged to be equivalent. We believe that our methods are useful for identity testing and also for comparative analysis of HOS of therapeutic monoclonal antibodies.

## Graphical abstract

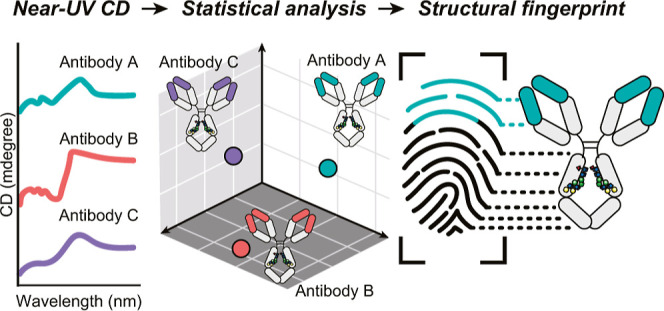

## Introduction

The higher-order structure (HOS) of therapeutic monoclonal antibody is one of the important quality characteristics.^1,2^ The integrity of HOS can be undermined by undesirable chemical alterations such as denaturation, fragmentation, and oxidation.^3,4^ Structural distortion in antibody molecules compromises the binding affinity for interacting molecules, such as antigen, Fc receptors, and complement, thus it impacts on its biological activity and also pharmacokinetics. Reduced bioactivity can diminish pharmacological effects and thus therapeutic efficacy. Moreover, misfolded/unfolded antibody molecules can form clusters via exposed hydrophobic regions and elicit insoluble aggregation. The aggregated particles can trigger immune response in patients.^5^ Therefore, the HOS of therapeutic monoclonal antibodies have profound correlation with both its efficacy and safety.

Biopharmaceutical industries use the information on the HOS of therapeutic monoclonal antibodies in many steps, including antibody-candidate selection, manufacturing process development, formulation development, and characterization. Traditionally, the HOS of therapeutic monoclonal antibodies has been assessed using low- to medium-resolution techniques including Fourier transform infrared spectroscopy, circular dichroism (CD) spectroscopy, intrinsic fluorescence spectroscopy, and differential scanning calorimetry.^2^ In addition, because the analysis of the obtained data has been performed by visual evaluation in many cases, an in-depth discussion of specificity, structural similarity, and statistical significance has not been undertaken. Moreover, given the increase in the development of new therapeutic monoclonal antibodies over the past two decades, the worldwide area of biosimilar development is rapidly expanding. Therefore, comparative structural analysis has become increasingly important. Biopharmaceutical industries should consider all relevant characteristics of the protein product to demonstrate that the biosimilar product is highly similar to the reference product.^6^ In recent years, regulatory authorities have increased their expectations for HOS assessment of biopharmaceuticals.^6^ Therefore, there is an increasing need for advanced, statistically comparable, and highly specific analytical methods for HOS of therapeutic monoclonal antibodies.

Near-UV CD has proven to be a pragmatic tool for the analysis of the HOS of proteins.^7–10^ However, the obtained spectra are not effortlessly used for statistical analysis or discussion. Recently, Oyama et al. addressed a statistical method for assessing the HOS of biopharmaceuticals.^11^ The study demonstrated that use of the Euclidean distance with the Savitzky–Golay noise reduction is effective for spectral distance assessment. Herein, we describe a method for HOS assessment of therapeutic monoclonal antibodies using a powerful combination of near-UV CD and statistical analysis. We obtained near-UV CD spectra of 14 therapeutic monoclonal antibodies, which are IgG1 antibodies that are currently in clinical use. Subsequently, the principal component analysis (PCA) was employed on the spectra to determine the discrimination capability of each HOS. In addition, we performed statistical tests, including equivalence test and Welch’s *t*-test to discuss their equivalency and statistically significant differences. These results clearly exemplify the advantage of the combinational use of near-UV CD and statistical analysis for the HOS assessment of therapeutic monoclonal antibodies.

## Materials and Methods

### Sample Preparations

Adalimumab (Humira), anifrolumab (Saphnelo), bevacizumab (Avastin), burosumab (Crysvita), daratumumab (Darzalex), durvalumab (Imfinzi), infliximab (Remicade), ipilimumab (Yervoy), necitumumab (Portrazza), ofatumumab (Kesimpta), omalizumab (Xolair), rituximab (Rituxan), trastuzumab (Herceptin), and ustekinumab (Stelara) were purchased from Japanese pharmaceutical distributors. Antibody samples were prepared at a concentration of 5 mg/mL by dilution with the formulation buffer of each antibody. A list of each formulation buffer is represented in Table S1.

### Near-UV Circular Dichroism Spectra

Near-UV CD spectra were obtained using a J-1500 CD spectrophotometer equipped with a PTC-510 Peltier thermostated cell holder (Jasco, Tokyo, Japan). The sample volume was 130 μL. The samples were scanned from 340 to 250 nm at a scanning speed of 20 nm/min. The measurements were performed at an ambient temperature (25 °C) by using cuvettes with a path length of 1.0 mm. The data acquisition interval was 0.1 nm, the bandwidth was 1 nm, and the response time was 4.0 s. Three scans were accumulated in one measurement. The measurements were performed in quintuplicate, and the buffer blanks were subtracted prior to data analysis. The UV absorbance at 280 nm was measured simultaneously with the near-UV CD spectra. The spectra were not smoothed.

### Principal Component Analysis

The near-UV CD spectra were offset-corrected to zero of the CD value at 340 nm and projected onto the principal component (PC) scores by taking the inner product with the eigenvectors (spectra of the PC) of their covariance matrix. Three significant PC scores were plotted. The analysis was performed by using scikit-learn 0.24.2 software.

### Quantitative Analysis of Spectral Difference and Their Comparison by Statistical Tests

The near-UV CD spectra were converted to weighted Euclidean distances, which reflect spectral differences. The equivalence tests and Welch’s *t*-tests (statistical significance tests) were performed using JWQHOS-531 software (JASCO Corporation, Tokyo, Japan). A one-to-one equivalence comparison was performed using one antibody as a reference and the remaining antibodies as test samples. Before conversion to the weighted Euclidean distance, the near-UV CD spectra were offset-corrected to zero of the mean CD value from 340 to 338 nm. In addition, the spectra were normalized by the absorbance at 280 nm simultaneously measured with CD spectrum to correct for variations of spectral intensity caused by dilution error. The weighted Euclidean distance between the mean spectrum of the reference $\overset{®}{r\left(\lambda \right)}$ and each spectrum of the reference ***r*** (λ) or test sample ***s*** (λ) was calculated using eq 1, where ***x*** (λ) takes ***r*** (λ) or ***s*** (λ). Wavelength-dependent weighting was performed by calculating the inner product of the squared vector of spectral differences and weight vector ω­(λ). The weight vector ω­(λ) was calculated using eq 2 based on the standard deviation vector **σ**(λ) of noise in the CD spectrum.^11^ The standard deviation vector **σ**(λ) was obtained from the high-tension (HT) voltage vector ***v*** (λ) of the photomultiplier tube acquired simultaneously with the CD spectrum using eq 3. When calculating the distance of ***r*** (λ)_*k*_, $\overset{®}{r\left(\lambda \right)}$ does not include ***r*** (λ)_*k*_ itself using eq 4 to avoid bias. On the other hand, when calculating the distance of ***s*** (λ), $\overset{®}{r\left(\lambda \right)}$ was calculated using all ***r*** (λ) using eq 5. To assume a normal distribution for the calculated weighted Euclidean distances *E*_r_ and *E*_s_, the *t*-value for the equivalence test was calculated using eq 6, where $\overset{®}{E_{r}\;}$ denotes the mean of the Euclidean distances of the reference, $\overset{®}{E_{s}\;}$ denotes the mean of the Euclidean distances of the test sample, σ_r_ and σ_s_ are the unbiased standard deviations of the reference and test sample, *m* and *n* are the size of the reference and test sample, and the equivalence margin δ was set to 2σ_r_.^12^ The *t*-value for Welch’s *t*-test was calculated using eq 7, where the meaning of each term in the equation is the same as in the equivalence test, but δ is not included in the equation.

In the comparison scheme, we judged the equivalence/significant difference based on the comparison of the Euclidean distance of the sample and the reference. In the equivalence test, the *p*-value was calculated on the left side of the *t*-distribution. This is because the Euclidean distance is the distance from the mean spectrum of the references, thus zero of the Euclidean distance means the spectrum is equal to reference spectrum itself. Therefore, the right side of *t*-distribution cannot be verified. Thus, a one-sided test is applied in this scheme. In Welch’s *t*-test, the *p*-value was calculated on the right of the *t*-distribution.

1 $$E=\sqrt{\frac{1}{s}\sum_{{i=\lambda }_{1}}^{\lambda_{s}}\left\{{\left(x_{i}-{\mathrm{r̅}}_{i}\right)}^{2}\omega_{i}\right\}}$$

2 $$\omega_{i}=\frac{\frac{1}{s}\sum_{{i=\lambda }_{1}}^{\lambda_{s}}\left|\sigma_{i}\right|}{\left|\sigma_{i}\right|}$$

3 $$\log_{10}\left(\sigma_{i}\right)=1.3313\times {\left(\log_{10}\left(v_{i}\right)\right)}^{2}-3.5163\times \log_{10}\left(v_{i}\right)-0.137$$

4 $$\overset{®}{r\left(\lambda \right)}=\frac{1}{m}\left[{r\left(\lambda \right)}_{1}+{r\left(\lambda \right)}_{2}+\cdot \cdot \cdot +{r\left(\lambda \right)}_{k-1}+{r\left(\lambda \right)}_{k+1}+\cdot \cdot \cdot +{r\left(\lambda \right)}_{n-1}+{r\left(\lambda \right)}_{m}\right]$$

5 $$\overset{®}{r\left(\lambda \right)}=\frac{1}{m}\sum_{k=1}^{m}{r\left(\lambda \right)}_{k}$$

6 $$t=\frac{\overset{®}{E_{s}\;}-\overset{®}{E_{r}\;}-\delta }{\sqrt{\frac{\sigma_{s}^{2}}{m}+\frac{\sigma_{r}^{2}}{n}}}$$

7 $$t=\frac{\overset{®}{E_{s}\;}-\overset{®}{E_{r}\;}}{\sqrt{\frac{\sigma_{s}^{2}}{m}+\frac{\sigma_{r}^{2}}{n}}}$$

### Calculation of Percent Identity of Amino Acid Sequence

The percent identity matrix among the 14 therapeutic monoclonal antibodies was calculated using the align tools in the UniProt (https://www.uniprot.org/align).^13^

## Results

### Near-UV Circular Dichroism Spectra

We selected and purchased 14 therapeutic monoclonal antibodies, which are currently in clinical use. To focus on the intrinsic protein structural differences, we selected antibodies that share the common IgG1 structure and are not engineered, such as antibody-drug conjugated, bispecific, and glycan-engineered antibodies. Anifrolumab was the only exception because the amino acid sequence of the constant region of the H chain was mutated (L234F, L235E, and P331S). The measured near-UV CD spectra are shown in Figure 1. The mean spectra with standard deviation and HT voltage are shown in Supporting Information Figure S1. The results clearly showed that each antibody has a unique near-UV CD spectrum. Although the averaged percent identity of amino acid sequence among these 14 antibodies are high (H chain, 88.9%; L chain, 86.3%), the shapes of the spectra were quite different (Tables S2 and S3). This is an outstanding observation from this study. In the near-UV region, the major chromophores contributing to CD spectra are disulfide bonds and aromatic amino acid residues, including tryptophan, tyrosine, and phenylalanine. Each of these chromophores contributes to the spectra at different ranges in the near-UV region, thus the spectra at each range gives structural interpretations separately.^4,14,15^

**Figure 1:**
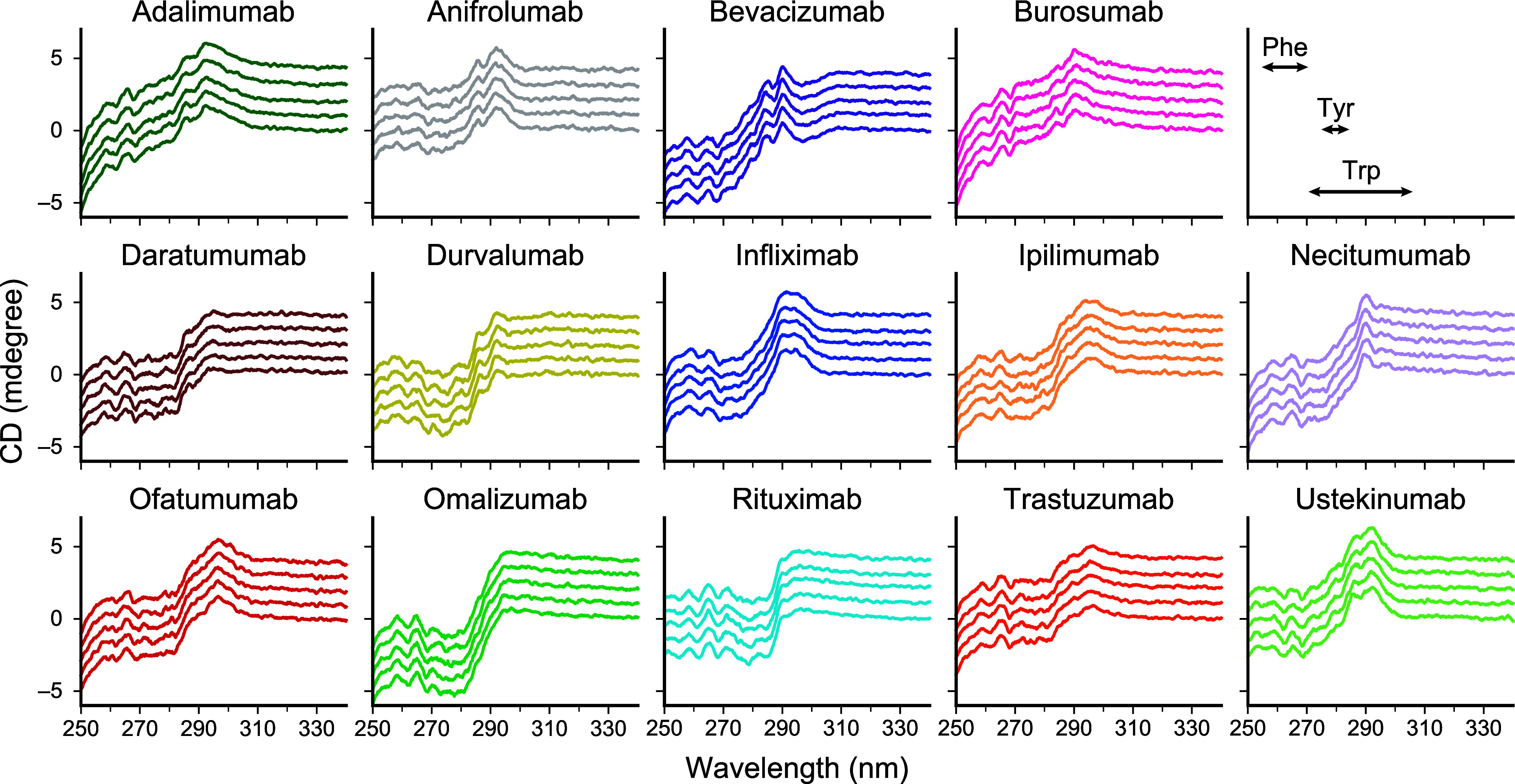
Obtained near-UV CD spectra of 14 antibodies. The near-UV CD spectra of adalimumab (dark green), anifrolumab (gray), bevacizumab (purple), burosumab (pink), daratumumab (brown), durvalumab (khaki), infliximab (blue), ipilimumab (sandy brown), necitumumab (lavender), ofatumumab (red), omalizumab (lime green), rituximab (cyan), trastuzumab (orange), and ustekinumab (pale green) are shown. The five replicate spectra of each antibody are displayed with a downward shift by 1 mdegree. On the top right, the regions correspond to Phe, Tyr, and Trp residues are highlighted.

The spectra of infliximab, ipilimumab, ofatumumab, and ustekinumab showed a positive and gentle peak at 290 nm. Interestingly, the shapes and magnitudes of the peaks at 290 nm were antibody-specific. The spectra from 305 to 270 nm are sensitive to tryptophan residues.^7,14,16,17^ Thus, these spectral differences could reflect the tertiary structure around the tryptophan residues.

The gradient decreases from 290 to 270 nm were also antibody-specific. The spectra in the range from 280 to 270 nm of daratumumab, durvalumab, ipilimumab, ofatumumab, omalizumab, and trastuzumab are relatively flat. The spectra from 282 to 275 nm are sensitive to tyrosine residues.^14^ Thus, these spectral differences could reflect the tertiary structure around the tyrosine residues.

The spectra of adalimumab and burosumab showed a significant decrease in the range from 270 to 250 nm. On the contrary, the spectra in the range of anifrolumab, bevacizumab, rituximab, and ustekinumab were flat. Generally, phenylalanine residues contribute to the spectra less than 270 nm.^14^ Thus, these spectral differences could reflect the tertiary structure around the phenylalanine residues.

The overall spectra of adalimumab and burosumab are quite similar based on visual observation.

The spectra of the formulation buffer of each antibody are shown in Figure S2. The magnitudes (amplitude of the spectrum between top and bottom) of these spectra were smaller than 0.8 mdegree, and no specific signals derived from additives such as amino acids were observed. The spectra of formulation buffer were subtracted from that of samples. Thus, the effects of formulation buffer on the spectrum of each antibody were negligibly small.

### Principal Component Analysis

PCA was employed to the obtained spectra to discriminate each HOS. The multidimensional data of approximately 900 data points in a single spectrum were reduced to 3D, that is, the PC. The results of PCA are shown in Figure 2. The origin of the graph represents the point with the mean of all the plots. The figure clearly shows that the plots of antibodies are dispersed separately. Thus, it is safe to note that the therapeutic monoclonal antibodies are identifiable using near-UV CD spectra and PCA. The explained variance of PC 1, 2, and 3 was 0.719, 0.148, and 0.062, respectively. The cumulative explained variance ratio was 0.929. The value means that 92.9% of the variation is explained by the three components. In other words, 92.9% of the original information is contained in these plots. The 7.1% of the information (variation) was lost during the transformation. The high cumulative explained variance ratio and also supports the suggestion that our method can discriminate each HOS of the antibodies.

**Figure 2:**
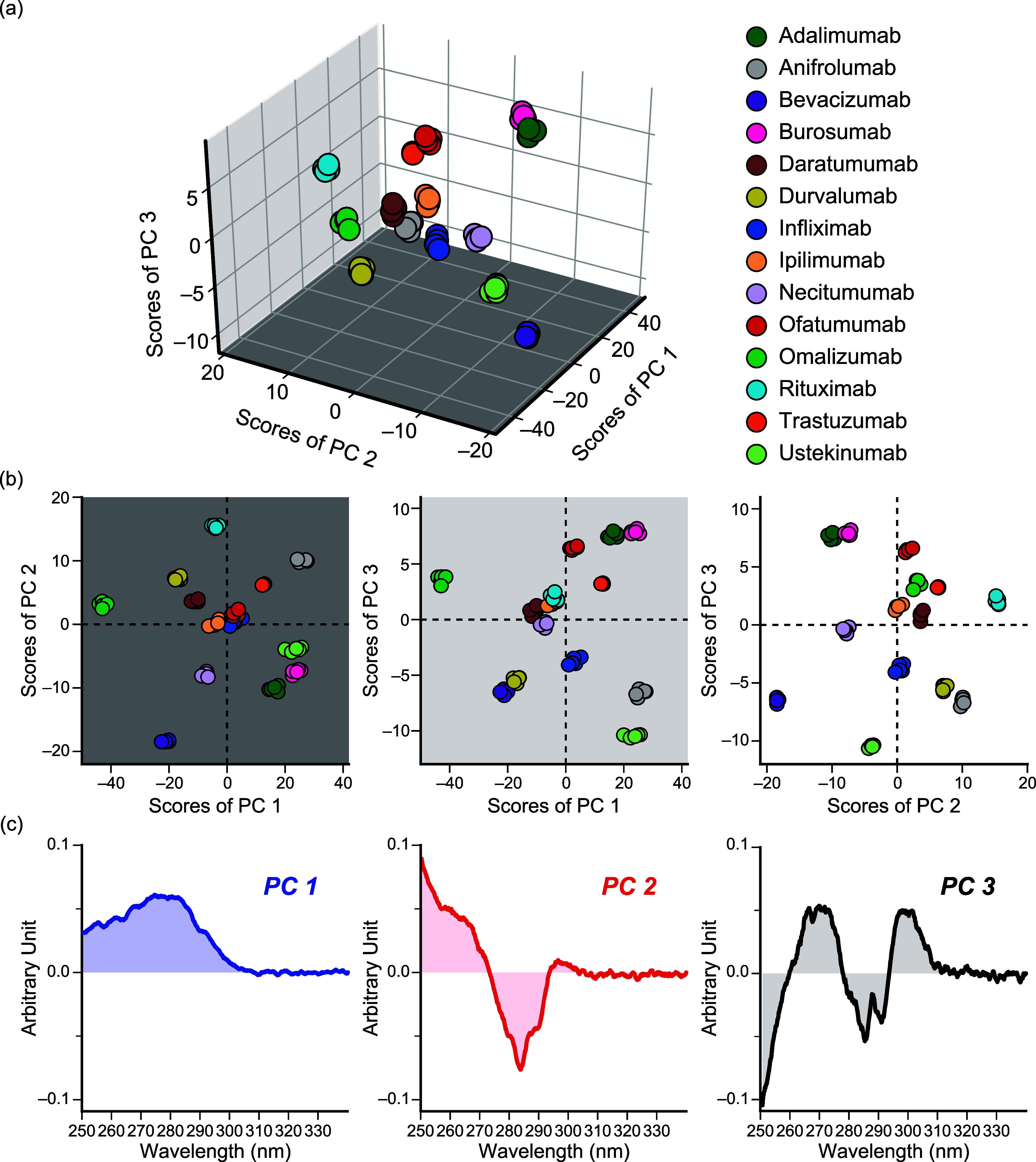
PCA of the near-UV CD spectra. (a) 3D scatter plot is shown. The 3 axes are PC 1, 2, and 3, respectively. (b) 2D scatter plots are shown. (c) The spectral data of PC 1, 2, and 3 are shown.

The plots of adalimumab and burosumab were similar in the score plots, which was consistent with the visual observation that their spectra were quite similar.

The spectra of PCs 1, 2, and 3 are shown in Figure 2c. The shapes of the spectra of PC 1, 2, and 3 were quite component-specific. The spectrum of PC 1 showed a positive and broad peak at 280 nm. The spectrum of PC 2 showed a negative peak at 285 nm. The spectrum of PC 3 showed two positive peaks at 270 and 300 nm, and a negative peak at 285 nm.

### Quantitative Analysis of Spectral Difference and Their Comparison by Statistical Tests

We performed statistical tests, including equivalence tests and statistical significance tests (Welch’s *t*-test). Prior to the statistical tests, the measured spectral data were transformed into the normalized and weighted Euclidean distances using JWQHOS-531 software. The overview of the procedure is illustrated in Figure 3. The measured Euclidean distances between each reference and the samples are shown in Figure 4. The higher values of the Euclidean distance indicate larger differences between sample spectra and reference spectra.

**Figure 3:**
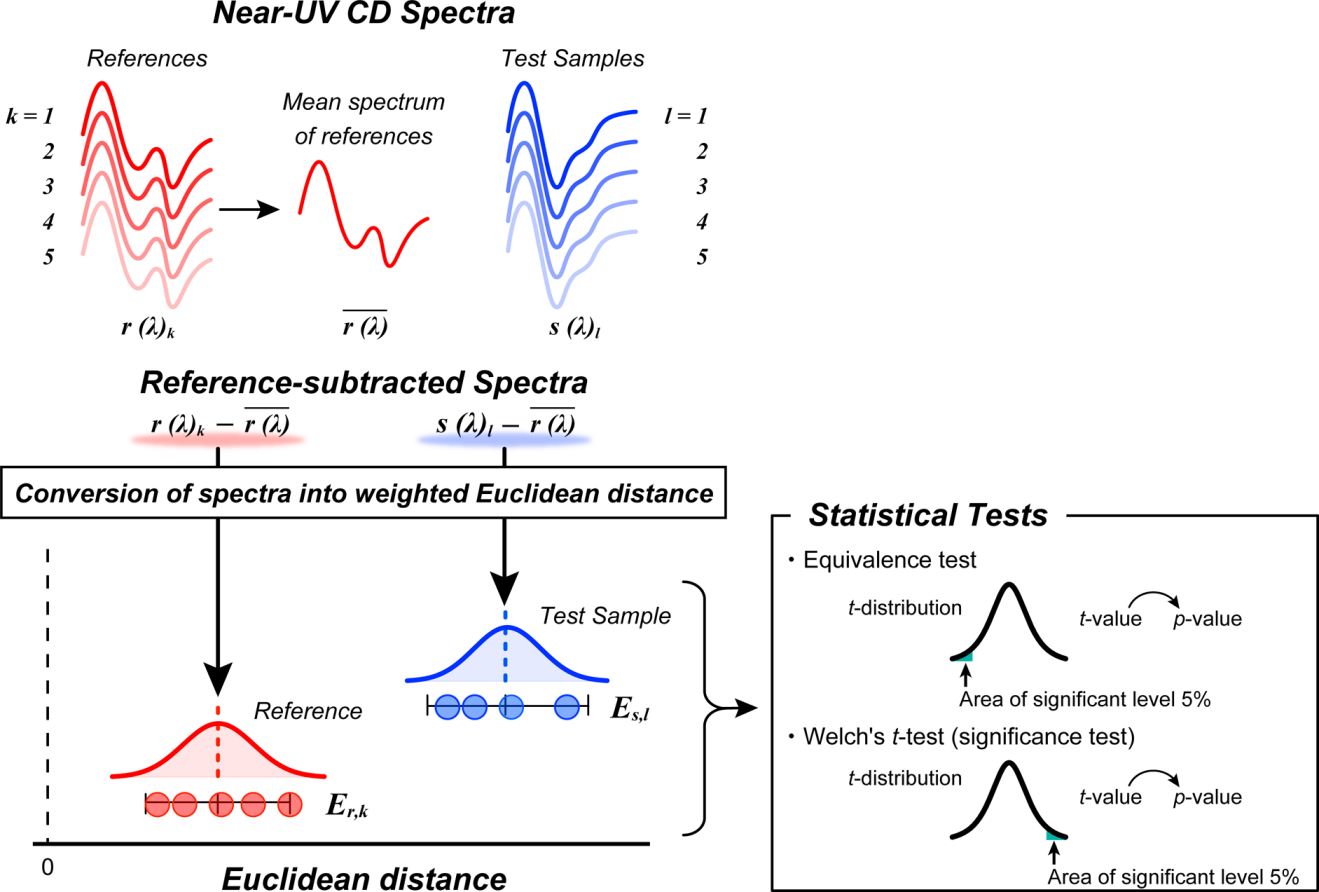
Schematic diagram of conversion of near-UV CD spectra into weighted Euclidean distance toward statistical test. The measured near-UV CD spectra of references (colored in red) and test samples (colored in blue) are represented as ***r*** (λ) and ***s*** (λ). The mean spectrum of reference was yielded by average of the five reference spectra (represented as $\overset{®}{r\left(\lambda \right)}$ ). The data of Euclidean distance were used for the statistical tests.

**Figure 4:**
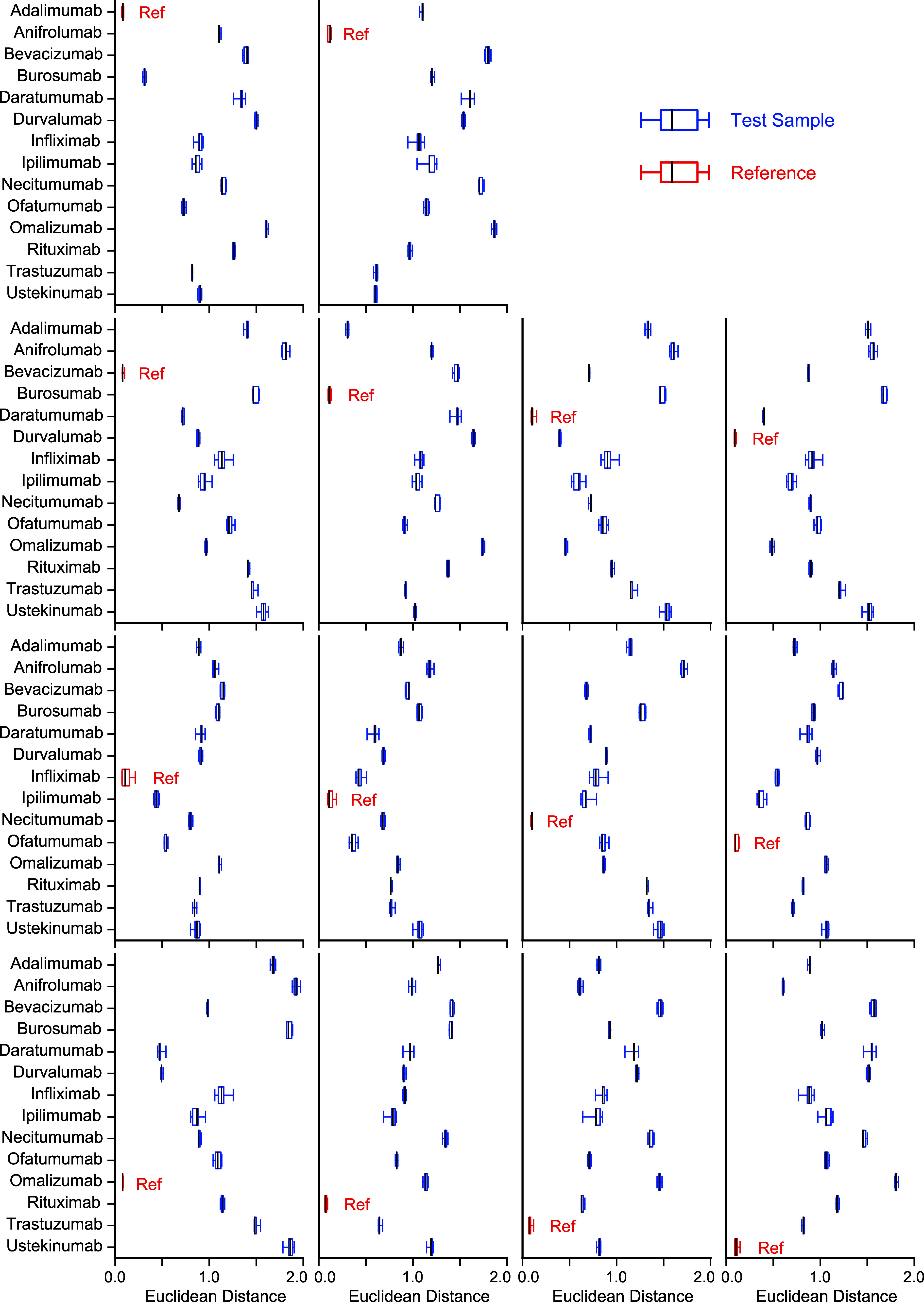
Quantified Euclidean distance between the reference and samples. Box-and-whisker plots of Euclidean distance are represented. The samples used as a reference are colored in red. The samples used as a test sample are colored in blue. The median values are shown in black line. The CD spectra of 14 antibodies were measured, and 14 analyses were performed by changing a reference antibody.

The equivalence test was performed on the obtained Euclidean distances. The null hypothesis was that the reference and sample were not equivalent. The *p*-values of the equivalence test are shown in Table S4. The *p*-value of almost all the samples was 1.0000, with three exceptions. The *p*-value of the comparison between infliximab (reference) and ipilimumab (test sample) was 0.9996, that of ofatumumab (test sample) was 0.9999, and that of ipilimumab (reference) and ofatumumab (test sample) was 0.9999. These exceptions were due to the higher variance in the repeatability of infliximab and ipilimumab. Overall, the null hypothesis was not rejected. The results demonstrate that the references were not equivalent to the samples.

Welch’s *t*-test was performed on the obtained Euclidean distances. The null hypothesis was that there is no statistical significance between the reference and the sample. The *p*-values of Welch’s *t*-test are shown in Table S5. The *p*-value of all of the samples was 0.0000. Thus, the null hypothesis was rejected. The results demonstrated a statistical significance between the reference and the samples. Contrary to our expectation, the *p*-value of the two antibodies of which the spectra are quite similar by visual assessment (i.e., adalimumab and burosumab) was 0.0000.

In addition, we performed the equivalence tests on the same antibodies but different production lots to determine whether our method could judge these antibodies to be equivalent (Figure 5). The equivalence test was employed to the Euclidean distances of 3 different lots of adalimumab, bevacizumab, rituximab, and trastuzumab. The results showed that all the *p*-values between the reference and test sample were lower than the significance level of 0.05. The results demonstrated that our method can judge whether the near-UV CD spectra of these different lot antibodies are equivalent.

**Figure 5:**
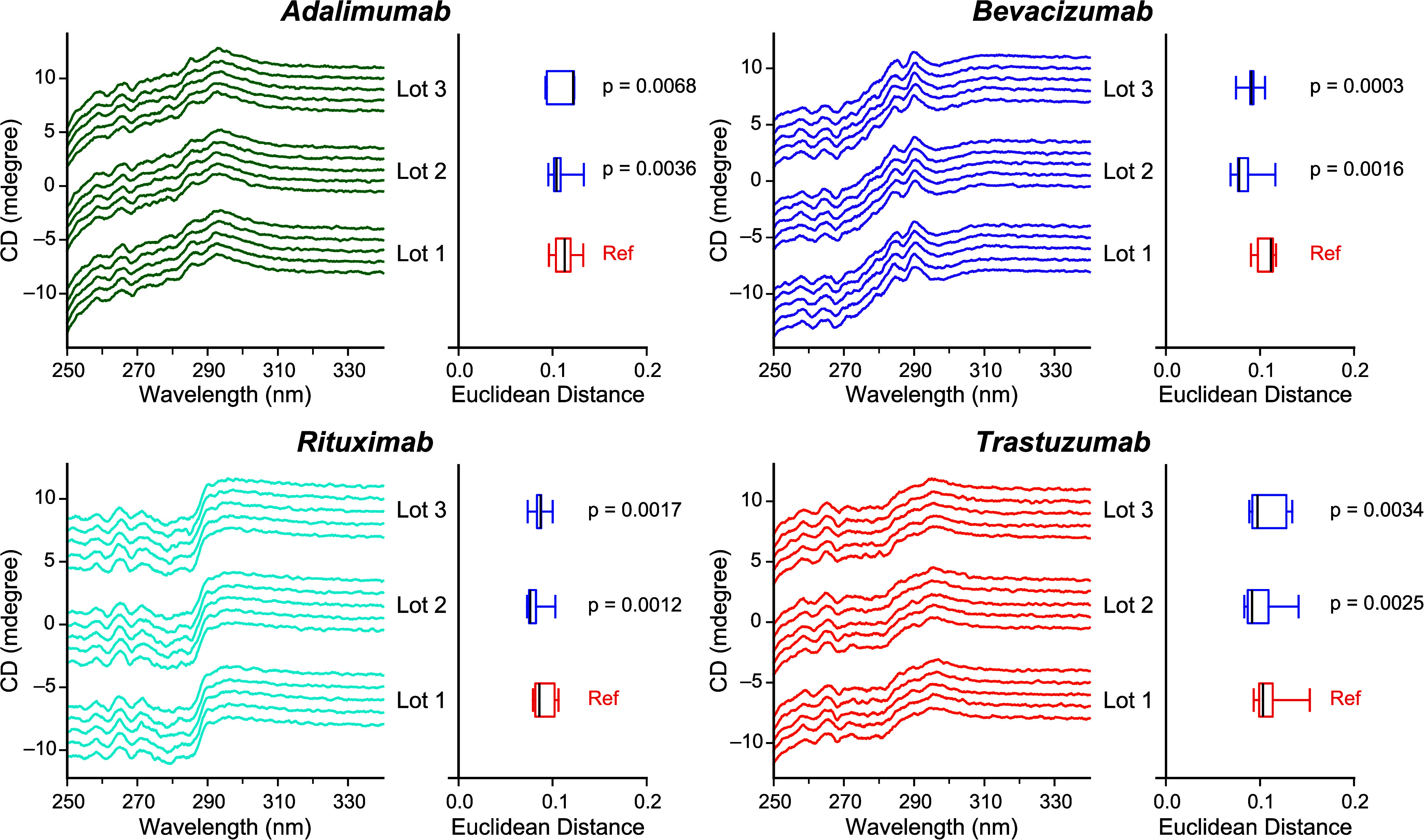
Equivalence tests on different production lots using Euclidean distance. The near-UV CD spectra of three lots of adalimumab (dark green), bevacizumab (purple), rituximab (cyan), and trastuzumab (orange) are shown (left). The 5 spectra were displayed with a downward shift by 1 mdegree. The box-and-whisker plots of the Euclidean distance are represented (right). The samples of lot 1, used as a reference, are colored in red. The samples of lot 2 and 3, used as a test sample, are colored in blue. The median values are shown in black line. The *p*-values of the equivalence test were represented on the right side of the box-and-whisker plot.

## Discussion

In recent years, several studies have focused on the structural fingerprints of therapeutic monoclonal antibodies. Specifically, nuclear magnetic resonance (NMR) is widely used in this field. Tokunaga et al. developed a novel NMR method to assess the structure of ^15^N-labeled and recombinant antibody.^18,19^ This technique was able to discriminate the heterogeneity of Fc-galactosylation. Hodgson et al. proposed an approach to assess the HOS of therapeutic monoclonal antibodies.^20^ The papain-digested antibodies (Fab and Fc) were employed to assess their HOS in the context of comparative studies. Not only NMR, Hageman et al. performed statistical equivalence tests on HOS using hydrogen/deuterium exchange mass spectrometry (HDX-MS) and discussed their biosimilarity between infliximab and its biosimilars.^21^ However, the structural fingerprint of therapeutic monoclonal antibody using CD spectra has not yet been studied extensively. In addition, the combination of near-UV CD and statistical analysis has not been explored.

Surprisingly, the obtained near-UV CD spectra of 14 therapeutic monoclonal antibodies were quite antibody-specific and irrespective of their amino acid sequence similarity, highlighting the feasibility of the structural fingerprint of therapeutic monoclonal antibodies. PCA translated the spectral data into a scatter plot comprising three components. The plots corresponding to each antibody were separately dispersed. Despite the fact that (1) these antibodies share the common IgG1 structure, (2) the sequential diversity largely occurs at the complementary determining region (CDR), and (3) the sequence identity is quite high, the combination of near-UV CD and PCA possesses a capability to discriminate the HOS of antibodies.

The quantitative analysis of spectral difference enabled an advanced discussion of equivalence and statistical significance. Statistically, 14 different antibodies were judged not to be equivalent to each other and also judged to be significantly different from other antibodies. Furthermore, our method can judge that the identical antibodies (here, we used different lot antibodies) are equivalent. These data demonstrated that the method can be useful for the identity test in specifications of therapeutic monoclonal antibodies. So far, a comparison of chromatogram of peptide mapping, high-performance liquid chromatography (HPLC), capillary electrophoresis (CE), enzyme-linked immunosorbent assay (ELISA), and bioassay is widely used for identity tests of therapeutic monoclonal antibodies. To our knowledge, the HOS is not evaluated as an identity test. As stated in the International Conference on Harmonization (ICH) Q2 (R2) guideline, specificity is critical as a performance characteristic for identity test.^22^ Our results demonstrated that the method possesses the capability to discriminate the HOS of both different antibodies and identical antibodies, thus the method is applicable for identity test. When attempting to use this method as an identity test, it is also required to consider interference from substances in samples for ensuring specificity. In particular, the CD spectrum can be interfered by additives that exhibit CD, such as l-amino acids. In our study, the magnitude of the spectrum of the formulation buffer was small, and we subtracted the spectrum of formulation buffer from that of samples. Thus, we confirmed that there was no interference by additives in the final reportable spectrum. Therefore, we consider that our analytical procedure had specificity that was required for the identity test. As mentioned above, our analytical procedure could be applied for the characterization of antibodies and even for batch release testing. Compared to the analytical techniques commonly used for release testing, such as peptide mapping or the immunochemical method, our method offers several advantages of reducing human and time resources because our method requires no sample preparation procedure and no specialized reagents. Our results also demonstrated that the method can also be useful for comparative analysis between (1) a reference product and its biosimilar and (2) pre- and postchanges in the manufacturing process. From this perspective, detailed data on the degraded samples are required.

Several studies have reported attempts to use spectral data of therapeutic monoclonal antibodies for the identity test. Shukla et al. demonstrated that Raman spectroscopy in combination with PCA and a support vector machine learning algorithm could be successfully used for monoclonal antibody identification.^23^ Duan et al. suggested that the combination of Raman spectroscopy and PCA achieved the level of specificity required to distinguish different types of antibodies.^24^ Our approach is feasible even if the analyst is not a specialist in statistics or machine learning. Moreover, CD is a widely used and noninvasive method, and small amount of the sample is required for measurement. Thus, we postulate that our approach is highly applicable for the quality control of therapeutic monoclonal antibodies.

Meanwhile, we could not answer the question of what types of HOS the near-UV CD are detecting, or the correlation between the spectral difference and structural difference, or simply, the interpretations of the near-UV CD spectra. To consider the location of chromophores (Trp, Tyr, and Phe) in the structure of antibody molecules, we illustrated all Trp, Tyr, and Phe in the amino acid sequence of the antibodies (Figures S3 and S4). The number and location of the residues in the variable region are significantly different among the antibodies. These unique patterns (number, location in sequence, 3D structure, exposure to the solvent, and the surrounding hydrophobic environment) of chromophores may have been reflected in the unique near-UV CD spectra. However, no regularity was found between the aromatic position and the spectrum pattern. Andersson et al. discussed that the exposure of Trp residues to solvent dominantly affects near-UV CD spectra.^25^ We calculated and compared the buried surface area (BSA) of Trp residues using crystal structural data (PDB data). The statistical correlation between the spectra and BSA of Trp residues was not observed by our analysis (data not shown). A detailed study from these perspectives remains elusive and should be explored in future works.

In conclusion, the powerful combination of near-UV CD spectra and statistical analysis is useful for HOS assessment of therapeutic monoclonal antibodies. The obtained fingerprints of the antibodies are quite important examples for scientists in the field of therapeutic monoclonal antibodies. We believe that this novel method is also widely applicable in many situations of biopharmaceutical development.

## Supplementary Material



## Notes

Mean near-UV CD spectra and HT voltage plots; spectra of formulation buffer of each antibody; amino acid sequence of H chain and L chain of the antibodies; formulation buffer of the antibodies; calculated percent identity of the H chain and L chain of the antibodies; and p-values of the equivalence test and Welch’s *t*-test (PDF)
