# Supplementary material for: The Structural Fingerprint of Therapeutic Monoclonal Antibodies Determined Using a Combination of Near-UV Circular Dichroism and Statistical Approach for Comparative Analysis
Source: Anal Chem. 2025 Jun 10;97(24):12578–86. doi: 10.1021/acs.analchem.5c00718 (PMC12199232; doi:10.1021/acs.analchem.5c00718)
Supplement: Supplementary file 1 [file ac5c00718_si_001.pdf]

Supporting Information

Structural fingerprint of therapeutic antibodies determined using a combination of near-UV CD and statistical approach for comparative analysis

Masato Kiyoshi[†,‡], Taiji Oyama[§,‡], Hiroko Shibata[†*], Satoko Suzuki[§], Yuji Higuchi[§], Kouhei Tsumoto[⁋,#], and Akiko Ishii-Watabe[†]

[†]Division of Biological Chemistry and Biologicals, National Institute of Health Sciences, 3-25-26 Tonomachi, Kawasaki-ku, Kawasaki, Kanagawa, 210-9501, Japan. [§]JASCO Corporation, 2967-5 Ishikawamachi, Hachioji, Tokyo, 192-8537, Japan, [⁋]Department of Bioengineering, School of Engineering, The University of Tokyo, 7-3-1 Hongo, Bunkyo-ku, Tokyo, 113-8656, Japan. [#]The Institute of Medical Science, The University of Tokyo, 4-6-1 Shirokanedai, Minato-ku, Tokyo, 108-8639, Japan.

Table of Contents

Figure S1: The mean near-UV CD spectra and HT voltage plots
Figure S2: The spectra of formulation buffer of each antibody
Figure S3: The amino acid sequence of H chain of the antibodies
Figure S4: The amino acid sequence of L chain of the antibodies
Table S1: The formulation buffer of the 14 therapeutic monoclonal antibodies
Table S2: The calculated percent sequence identity of the H chain of the 14 therapeutic monoclonal antibodies
Table S3: The calculated percent sequence identity of the L chain of the 14 therapeutic monoclonal antibodies
Table S4: The p-values of the equivalence test
Table S5: The p-values of the Welch's t-test.

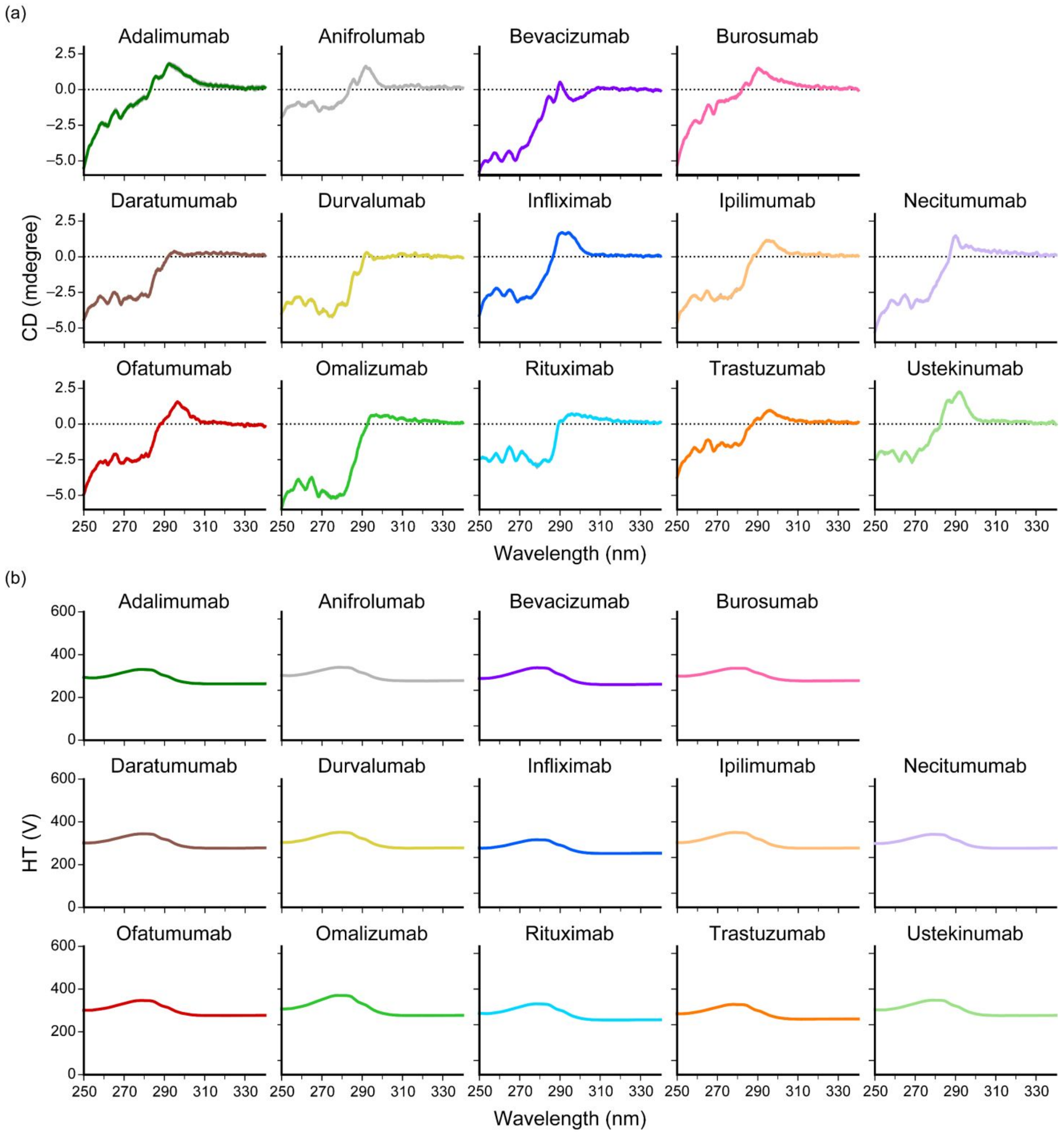

Figure S1. (a) The mean near-UV CD spectra of adalimumab (dark green), anifrolumab (grey), bevacizumab (purple), burosumab (pink), daratumumab (brown), durvalumab (khaki), infliximab (blue), ipilimumab (sandy brown), necitumumab (lavender), ofatumumab (red), omalizumab (lime green), rituximab (cyan), trastuzumab (orange), and ustekinumab (pale green) are shown. The standard deviation of five measurements on each sample is shown in light gray. (b) The HT voltage plots of the measurements.

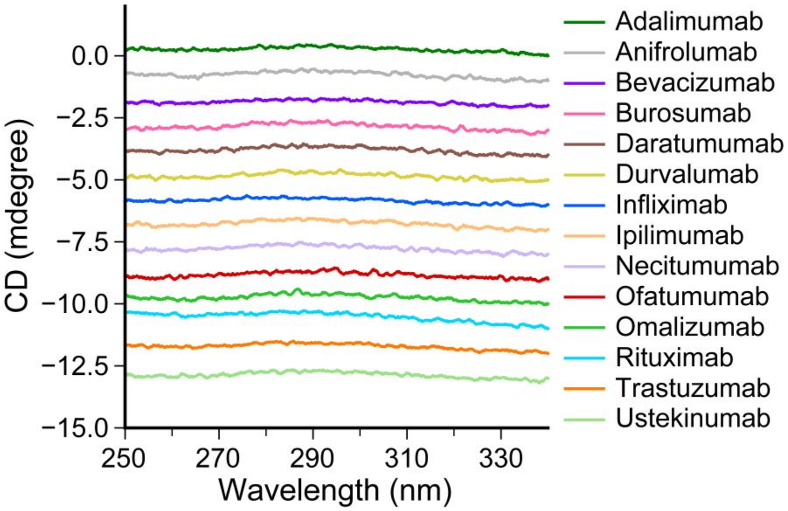

Figure S2. The spectra of formulation buffer of each antibody. The near-UV CD spectra of formulation buffer of adalimumab (dark green), anifrolumab (grey), bevacizumab (purple), burosumab (pink), daratumumab (brown), durvalumab (khaki), infliximab (blue), ipilimumab (sandy brown), necitumumab (lavender), ofatumumab (red), omalizumab (lime green), rituximab (cyan), trastuzumab (orange), and ustekinumab (pale green) are shown. The spectra were displayed with a downward shift by 1 mdegree.

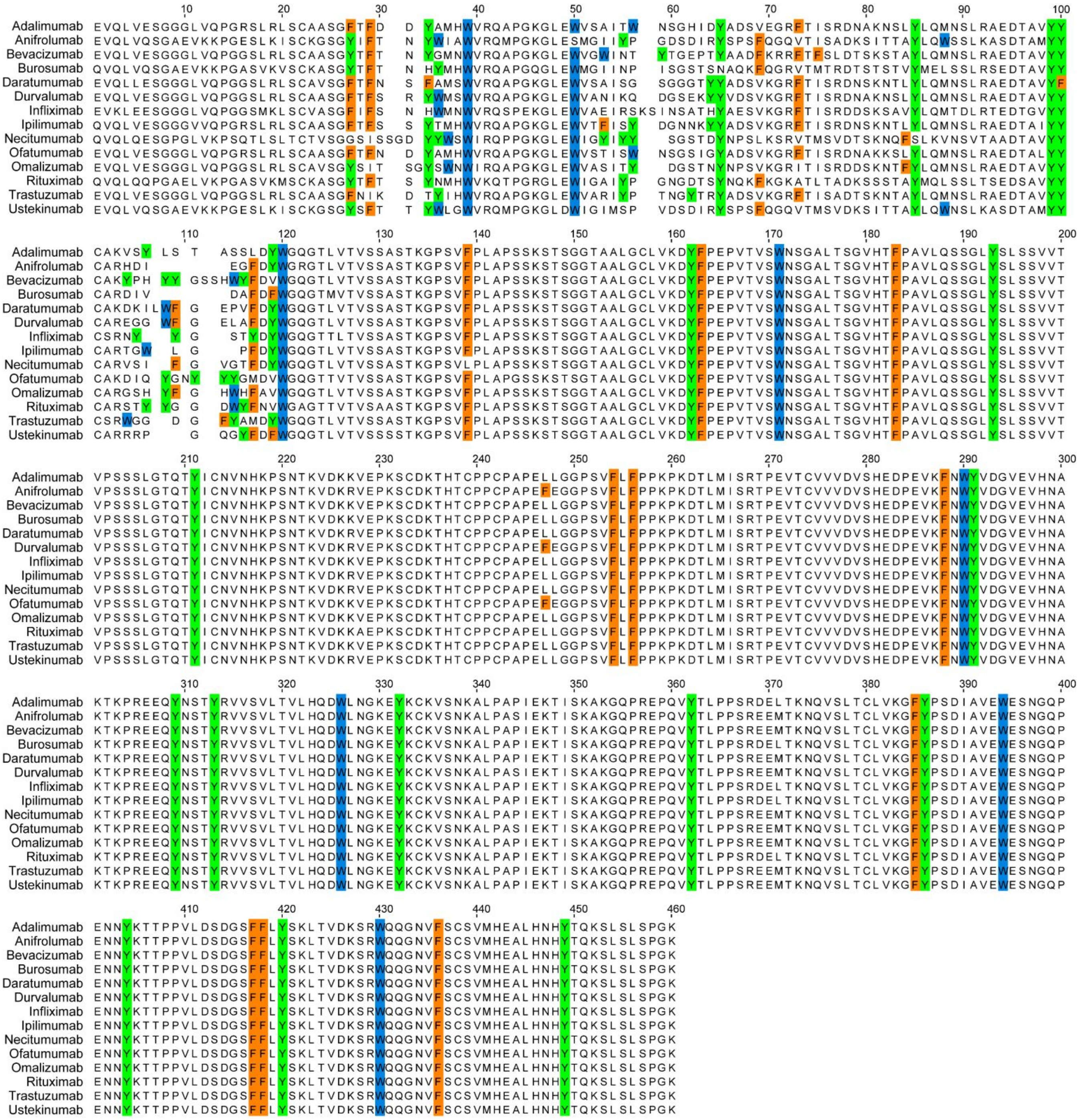

Figure S3. The amino acid sequence of H chain of the antibodies. The background of Trp is colored in blue, Tyr is colored in green, and Phe is colored in orange.

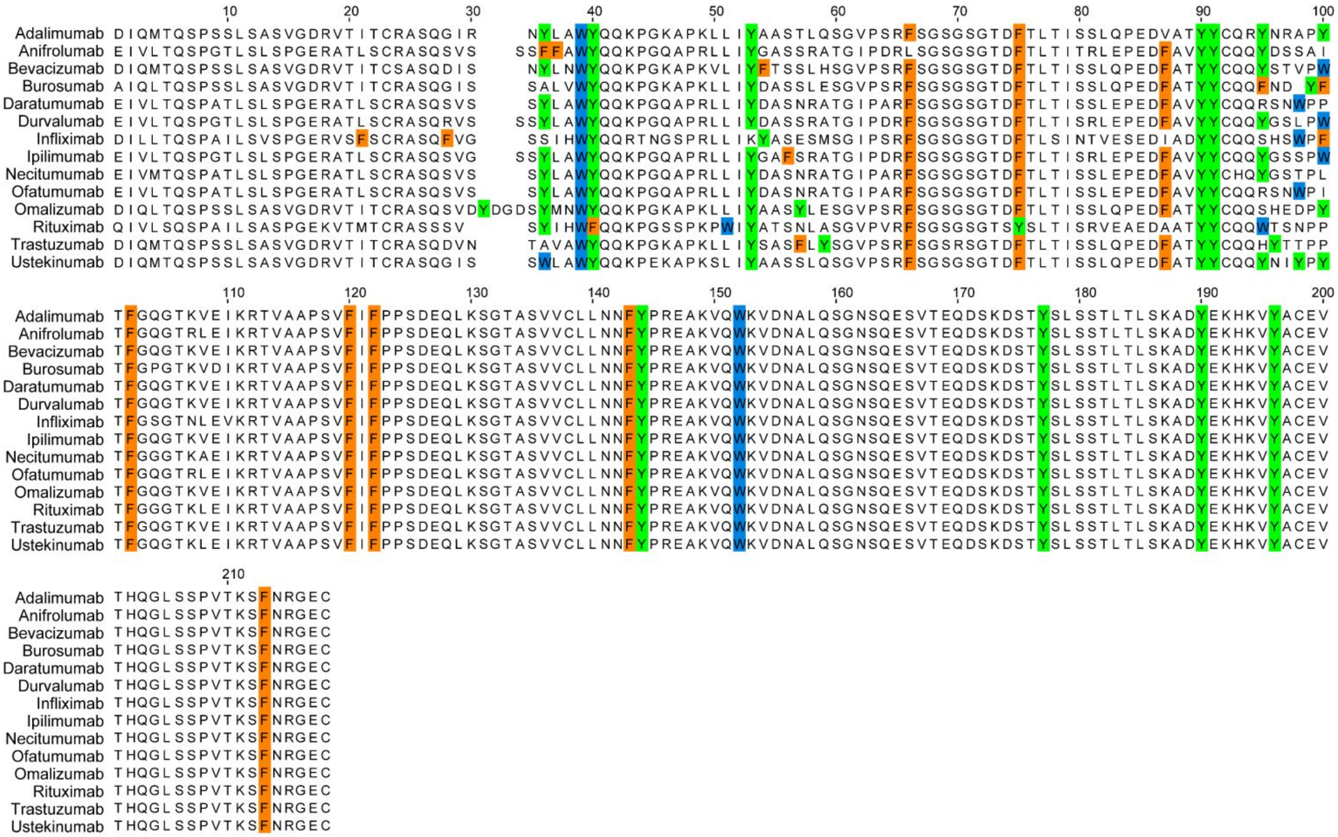

Figure S4. The amino acid sequence of L chain of the antibodies. The background of Trp is colored in blue, Tyr is colored in green, and Phe is colored in orange.

Table S1. The formulation buffer of the 14 therapeutic monoclonal antibodies.

Name	Additives	Quantity (mg)	Volume (ml)	Concentration		pH
Adalimumab	D-Mannitol	16.8	0.4	42.0	mg/ml	4.9～5.5
	Polysorbate 80	0.4		1.0	mg/ml	
Anifrolumab	L-histidine	3	2	1.5	mg/ml	5.4～6.4
	L-histidine hydrochloride	6		3.0	mg/ml	
	L-lysine hydrochloride	18		9.0	mg/ml	
	Trehalose hydrate	98		49.0	mg/ml	
	Polysorbate 80	1		0.5	mg/ml	
Bevacizumab*	Trehalose	960	16	60.0	mg/ml	5.9～6.3
	Sodium dihydrogen phosphate monohydrate	92.8		5.8	mg/ml	
	Sodium phosphate monobasic anhydrous	19.2		1.2	mg/ml	
	Polysorbate 20	6.4		0.4	mg/ml	
Burosumab	L-histidine	1.6	1	1.6	mg/ml	6.0～6.5
	Polysorbate 80	0.5		0.5	mg/ml	
	L-methionine	1.5		1.5	mg/ml	
Daratumumab	D-Mannitol	1275	5	255.0	mg/ml	5.3～5.8
	Sodium chloride	17.5		3.5	mg/ml	
	Sodium Acetate Hydrate	14.8		3.0	mg/ml	
	Polysorbate 20	2		0.4	mg/ml	
	Acetic acid	0.9		0.2	mg/ml	
Durvalumab	L-histidine	4.8	2.4	2.0	mg/ml	5.5～6.5
	L-histidine hydrochloride	6.5		2.7	mg/ml	
	Trehalose hydrate	250		104.2	mg/ml	
	Polysorbate 80	0.5		0.2	mg/ml	
Infliximab*	Sucrose	500	10	50.0	mg/ml	6.9～7.5
	Polysorbate 80	0.5		50.0	μg/ml	
	Sodium dihydrogen phosphate monohydrate	2.2		0.2	mg/ml	
	Disodium hydrogen phosphate dihydrate	6.1		0.6	mg/ml	
Ipilimumab*	Trometamol hydrochloride	12.6	4	3.2	mg/ml	6.6～7.6
	Sodium chloride	23.4		5.9	mg/ml	
	D-Mannitol	40		10.0	mg/ml	
	Diethylenetriaminepentaacetic acid	0.16		40	μg/ml	
	Polysorbate 80	0.44		110	μg/ml	
Necitumumab	Sodium Citrate hydrate	127.5	50	2.6	mg/ml	5.7～6.3
	Anhydrous citric acid	12.8		0.3	mg/ml	
	Glycine	499.2		10.0	mg/ml	
	Sodium chloride	116.9		2.3	mg/ml	
	D-Mannitol	455.5		9.1	mg/ml	
	Polysorbate 80	5		0.1	mg/ml	
Ofatumumab*	L-Arginine	4	0.4	10.0	mg/ml	5.3～5.7
	Sodium acetate Hydrate	2.72		6.8	mg/ml	
	Polysorbate 80	0.08		0.2	mg/ml	
	Disodium edetate hydrate	0.007		18	μg/ml	
Omalizumab	L-Arginine hydrochloride	21.05	0.5	42.1	mg/ml	5.8～6.2
	L-histidine hydrochloride hydrate	1.17		2.3	mg/ml	
	L-histidine	0.68		1.4	mg/ml	
	Polysorbate 20	0.2		0.4	mg/ml	
Rituximab*	Polysorbate 80	7	10	0.7	mg/ml	6.5±0.3
	Sodium chloride	90		9.0	mg/ml	
	Sodium Citrate Dihydrate	71.4		7.1	mg/ml	
	Anhydrous Citric Acid	1.4		140	μg/ml	
Trastuzumab	Trehalose hydrate	136.2	7.2	18.9	mg/ml	5.8～6.4
	L-histidine hydrochloride	3.36		0.5	mg/ml	
	L-histidine	2.16		0.3	mg/ml	
	Polysorbate 20	0.6		83.0	μg/ml	
Ustekinumab	Sucrose	2210	26	85.0	mg/ml	5.7～6.3
	L-histidine	20		0.8	mg/ml	
	L-histidine hydrochloride	27		1.0	mg/ml	
	Polysorbate 80	10.4		0.4	mg/ml	
	L-methionine	10.4		0.4	mg/ml	
	Disodium edetate hydrate	0.52		20	μg/ml	

* These formulations may include pH control additives and tonicity agents.

Table S2. The calculated percent sequence identity of the H chain of the 14 therapeutic monoclonal antibodies.

	Adalimumab	Anifrolumab	Bevacizumab	Burosumab	Daratumumab	Durvalumab	Infliximab	Ipilimumab	Necitumumab	Ofatumumab	Omalizumab	Rituximab	Trastuzumab	Ustekinumab
Adalimumab	100													
Anifrolumab	86.32	100												
Bevacizumab	91.35	87.44	100											
Burosumab	88.09	88.54	88.79	100										
Daratumumab	93.35	86.77	91.37	87.42	100									
Durvalumab	92.67	88.34	90.91	86.71	94.01	100								
Infliximab	90.34	85.33	89.71	87.13	89.69	89.91	100							
Ipilimumab	93.96	87.42	91.29	88.74	94.42	94.64	90.11	100						
Necitumumab	87.7	85.84	87.08	85.65	88.17	87.7	84.98	88.09	100					
Ofatumumab	93.13	85.87	89.82	86.07	91.15	91.57	87.89	91.07	84.82	100				
Omalizumab	91.52	87.42	92.89	87.19	91.98	91.52	89.49	91.48	89.53	89.53	100			
Rituximab	85.94	87.02	87.33	90.83	85.3	84.6	85.65	86.55	84.41	83.52	86.19	100		
Trastuzumab	92.43	88.09	92	88.49	92.67	92.44	90.81	92.62	89.01	90.67	93.06	87.02	100	
Ustekinumab	86.35	94.63	87.95	88.76	86.83	86.83	84.94	87.67	86.8	84.82	87.92	86.64	87.47	100

Table S3. The calculated percent sequence identity of the L chain of the 14 therapeutic monoclonal antibodies.

	Adalimumab	Anifrolumab	Bevacizumab	Burosumab	Daratumumab	Durvalumab	Infliximab	Ipilimumab	Necitumumab	Ofatumumab	Omalizumab	Rituximab	Trastuzumab	Ustekinumab
Adalimumab	100													
Anifrolumab	81.31	100												
Bevacizumab	92.99	81.31	100											
Burosumab	91.55	82.63	91.55	100										
Daratumumab	84.58	92.06	84.58	85.92	100									
Durvalumab	84.58	94.42	85.05	85.45	95.33	100								
Infliximab	78.04	80.84	77.10	78.87	81.78	81.31	100							
Ipilimumab	84.11	94.88	84.11	84.04	94.39	97.67	81.31	100						
Necitumumab	84.11	91.59	83.64	84.04	95.79	94.86	80.84	93.93	100					
Ofatumumab	83.64	93.46	83.64	84.98	98.60	94.39	82.24	93.46	95.33	100				
Omalizumab	92.99	82.79	92.06	92.96	86.45	85.12	80.84	85.12	84.58	85.51	100			
Rituximab	80.75	80.28	80.75	80.66	82.63	81.69	79.81	81.69	82.16	82.16	83.10	100		
Trastuzumab	92.06	81.78	92.06	91.08	85.05	84.11	78.50	83.64	84.11	83.64	92.52	80.75	100	
Ustekinumab	94.86	83.18	92.99	92.49	84.58	85.05	78.97	84.11	84.58	84.58	92.52	81.22	91.59	100

Table S4. The *p*-values of the equivalence test.

		Reference													
		Adalimumab	Anifrolumab	Bevacizumab	Burosumab	Daratumumab	Durvalumab	Infliximab	Ipilimumab	Necitumumab	Ofatumumab	Omalizumab	Rituximab	Trastuzumab	Ustekinumab
**Test Sample**	Adalimumab	N.D.	1.0000	1.0000	1.0000	1.0000	1.0000	1.0000	1.0000	1.0000	1.0000	1.0000	1.0000	1.0000	1.0000
	Anifrolumab	1.0000	N.D.	1.0000	1.0000	1.0000	1.0000	1.0000	1.0000	1.0000	1.0000	1.0000	1.0000	1.0000	1.0000
	Bevacizumab	1.0000	1.0000	N.D.	1.0000	1.0000	1.0000	1.0000	1.0000	1.0000	1.0000	1.0000	1.0000	1.0000	1.0000
	Burosumab	1.0000	1.0000	1.0000	N.D.	1.0000	1.0000	1.0000	1.0000	1.0000	1.0000	1.0000	1.0000	1.0000	1.0000
	Daratumumab	1.0000	1.0000	1.0000	1.0000	N.D.	1.0000	1.0000	1.0000	1.0000	1.0000	1.0000	1.0000	1.0000	1.0000
	Durvalumab	1.0000	1.0000	1.0000	1.0000	1.0000	N.D.	1.0000	1.0000	1.0000	1.0000	1.0000	1.0000	1.0000	1.0000
	Infliximab	1.0000	1.0000	1.0000	1.0000	1.0000	1.0000	N.D.	1.0000	1.0000	1.0000	1.0000	1.0000	1.0000	1.0000
	Ipilimumab	1.0000	1.0000	1.0000	1.0000	1.0000	1.0000	0.9996	N.D.	1.0000	1.0000	1.0000	1.0000	1.0000	1.0000
	Necitumumab	1.0000	1.0000	1.0000	1.0000	1.0000	1.0000	1.0000	1.0000	N.D.	1.0000	1.0000	1.0000	1.0000	1.0000
	Ofatumumab	1.0000	1.0000	1.0000	1.0000	1.0000	1.0000	0.9999	0.9999	1.0000	N.D.	1.0000	1.0000	1.0000	1.0000
	Omalizumab	1.0000	1.0000	1.0000	1.0000	1.0000	1.0000	1.0000	1.0000	1.0000	1.0000	N.D.	1.0000	1.0000	1.0000
	Rituximab	1.0000	1.0000	1.0000	1.0000	1.0000	1.0000	1.0000	1.0000	1.0000	1.0000	1.0000	N.D.	1.0000	1.0000
	Trastuzumab	1.0000	1.0000	1.0000	1.0000	1.0000	1.0000	1.0000	1.0000	1.0000	1.0000	1.0000	1.0000	N.D.	1.0000
	Ustekinumab	1.0000	1.0000	1.0000	1.0000	1.0000	1.0000	1.0000	1.0000	1.0000	1.0000	1.0000	1.0000	1.0000	N.D.

Table S5. The *p*-values of the Welch's *t*-test.

		Reference													
		Adalimumab	Anifrolumab	Bevacizumab	Burosumab	Daratumumab	Durvalumab	Infliximab	Ipilimumab	Necitumumab	Ofatumumab	Omalizumab	Rituximab	Trastuzumab	Ustekinumab
**Test Sample**	Adalimumab	N.D.	0.0000	0.0000	0.0000	0.0000	0.0000	0.0000	0.0000	0.0000	0.0000	0.0000	0.0000	0.0000	0.0000
	Anifrolumab	0.0000	N.D.	0.0000	0.0000	0.0000	0.0000	0.0000	0.0000	0.0000	0.0000	0.0000	0.0000	0.0000	0.0000
	Bevacizumab	0.0000	0.0000	N.D.	0.0000	0.0000	0.0000	0.0000	0.0000	0.0000	0.0000	0.0000	0.0000	0.0000	0.0000
	Burosumab	0.0000	0.0000	0.0000	N.D.	0.0000	0.0000	0.0000	0.0000	0.0000	0.0000	0.0000	0.0000	0.0000	0.0000
	Daratumumab	0.0000	0.0000	0.0000	0.0000	N.D.	0.0000	0.0000	0.0000	0.0000	0.0000	0.0000	0.0000	0.0000	0.0000
	Durvalumab	0.0000	0.0000	0.0000	0.0000	0.0000	N.D.	0.0000	0.0000	0.0000	0.0000	0.0000	0.0000	0.0000	0.0000
	Infliximab	0.0000	0.0000	0.0000	0.0000	0.0000	0.0000	N.D.	0.0000	0.0000	0.0000	0.0000	0.0000	0.0000	0.0000
	Ipilimumab	0.0000	0.0000	0.0000	0.0000	0.0000	0.0000	0.0000	N.D.	0.0000	0.0000	0.0000	0.0000	0.0000	0.0000
	Necitumumab	0.0000	0.0000	0.0000	0.0000	0.0000	0.0000	0.0000	0.0000	N.D.	0.0000	0.0000	0.0000	0.0000	0.0000
	Ofatumumab	0.0000	0.0000	0.0000	0.0000	0.0000	0.0000	0.0000	0.0000	0.0000	N.D.	0.0000	0.0000	0.0000	0.0000
	Omalizumab	0.0000	0.0000	0.0000	0.0000	0.0000	0.0000	0.0000	0.0000	0.0000	0.0000	N.D.	0.0000	0.0000	0.0000
	Rituximab	0.0000	0.0000	0.0000	0.0000	0.0000	0.0000	0.0000	0.0000	0.0000	0.0000	0.0000	N.D.	0.0000	0.0000
	Trastuzumab	0.0000	0.0000	0.0000	0.0000	0.0000	0.0000	0.0000	0.0000	0.0000	0.0000	0.0000	0.0000	N.D.	0.0000
	Ustekinumab	0.0000	0.0000	0.0000	0.0000	0.0000	0.0000	0.0000	0.0000	0.0000	0.0000	0.0000	0.0000	0.0000	N.D.
